# Utilizing Martian samples for future planetary exploration—Characterizing hazards and resources

**DOI:** 10.1073/pnas.2404251121

**Published:** 2025-01-06

**Authors:** Charles Whetsel, Joel S. Levine, Stephen J. Hoffman, Clare M. Luckey, Kevin D. Watts, Erik L. Antonsen

**Affiliations:** ^a^Moon to Mars Support Office, Planetary Sciences Directorate, Jet Propulsion Laboratory-California Institute of Technology, Pasadena, CA 91011; ^b^Department of Applied Science, William and Mary, Williamsburg, VA 23187; ^c^Engineering and Technology Group, The Aerospace Corporation, Houston, TX 77058; ^d^Exploration Mission Planning Office, Exploration Integration and Science Directorate, NASA Johnson Space Center, Houston, TX 77058; ^e^Division of Space, Ecological, Arctic, and Resource-limited (SPEAR) Medicine, Department of Emergency Medicine, Massachusetts General Hospital, Boston, MA 02114

**Keywords:** Mars, spaceflight, exploration

## Abstract

One of the most surprising and important findings of the first human landings on the Moon was the discovery of a very fine layer of lunar dust covering the entire surface of Moon along with the negative impacts of this dust on the well-being and operational effectiveness of the astronauts, their equipment, and instrumentation. The United States is now planning for human missions to Mars, a planet where dust can also be expected to be ubiquitous for many or most landing sites. For these missions, the design and operations of key hardware systems must take this dust into account, especially when related to crew health and safety. Improved understanding of Martian dust characteristics can inform its potential to also perform transport of microorganisms, both those inadvertently brought to Mars by the astronauts, or, if Martian microorganisms exist, the potential for their inadvertent return to Earth with the astronauts. Careful planning and design are needed to assure that future missions do not violate the United Nations Outer Space Treaty (1967) signed by all spacefaring nations. In this paper, we review the impact of lunar dust on the Apollo missions and identify several questions about dust in the atmosphere of Mars that may be answered by the curated samples that would be returned by the planned Mars Sample Return (MSR) Campaign. These answers would not only provide an opportunity to better understand the history of Mars but could also reduce uncertainty in charting the future of humanity’s exploration of the planet.

NASA is commencing the planning process for a Mars Sample Return (MSR) campaign of missions which would return samples collected by the Mars 2020 *Perseverance* Rover. The decision to implement these missions will not be finalized until NASA’s completion of the National Environmental Policy Act process. Among the areas in which the space science community would gain value from MSR are the ways in which returned samples will inform planning for the development and operations of future crewed missions to Mars. This value will be derived from two primary areas examined further in this paper: a) “ground truth” characterization of returned regolith, both chemical and mineral composition, as well as particle size distributions, and b) biological characterization in support of a well-considered Planetary Protection (PP) strategy for future Martian exploration. The importance to designers of future crewed missions in understanding and planning for the regolith/dust environment on Mars is underscored by lessons from the Apollo missions. These include both human health, safety, and comfort/performance considerations, as well as the design of hardware systems and operational utilization approaches. Additionally, studies of returned rocks and regolith may provide some insight whether these materials might eventually prove suitable as a construction material for future landing sites, trails, roads, or berms in the long-term human exploration of Mars. The importance of understanding the Martian dust environment, as informed by lessons from Apollo, as well as how this information would benefit planning for future missions is explored further in this paper (Fig. 1).

**Fig. 1.**
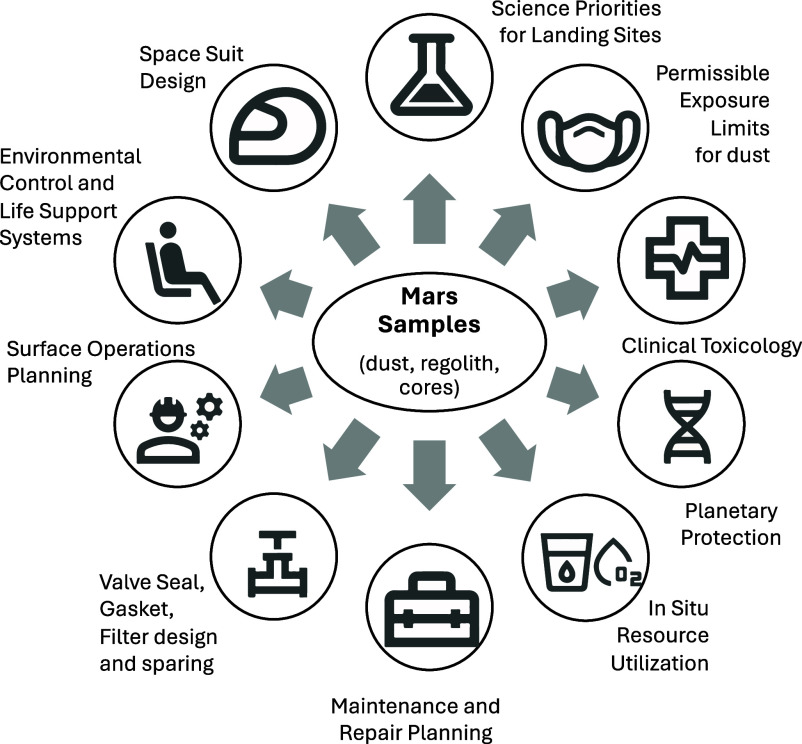
Knowledge gained from returned Mars Samples informs a diversity of domains for future exploration.

## Lunar Dust and the Apollo Mission

Although Earth’s Moon and Mars have evolved as the result of many different planetary processes, dust is a predominant feature across most terrains on both bodies. In addition, while Martian dust will not behave exactly like lunar dust in every case, there are many lessons from the Apollo experience with lunar dust that are instructive in understanding the kinds of unforeseen problems that may arise when the dust environment is not well characterized.

On 20 July 1969, as he was about to become the first human to set foot on another world, Apollo 11 astronaut Neil Armstrong, radioed the following message back to Earth (1):

*“I’m at the foot of the ladder. The LM footpads are only depressed in the surface about 1 or 2 inches, although the surface appears to be very, very fine-grained, as you get close to it, it’s almost like a powder; down there, it’s very fine... I’m going to step off the LM now. That’s one small step for [a] man: one giant leap for mankind. As the-The surface is fine and powdery. I can-I can pick it up loosely with my toe. It does adhere in fine layers like powdered charcoal to the sole and sides of my boots. I only go in a small fraction of an inch. Maybe an eighth of an inch, but I can see the footprints of my boots and the treads in the fine sandy particles.”*

Armstrong first encountered lunar dust during the landing of the Lunar Module (LM) on the lunar surface as the LM rocket exhaust gases blew surface dust into the thin lunar atmosphere. The “very, very fined-grained” dust observed by Armstrong would become a problem for all the Apollo astronauts that landed on the Moon. The lunar dust impacted astronaut health, the astronaut’s equipment, and instrumentation and lunar surface operations. Apollo 17 Astronaut Eugene Cernan reported the following in his postflight briefing (2):

*“Dust—I think probably one of the most aggravating, restricting facets of lunar surface exploration is the dust and its adherence to everything no matter what kind of material, whether it be skin, suit material, metal, no matter what it be and its restrictive friction-like action to everything it gets on. For instance, the simple large tolerance mechanical devices on the Rover began to show the effect of dust as the EVAs went on. By the middle or end of the third EVA, simple things like bag locks and the lock which held the pallet on the Rover began not only to malfunction but to not function at all. They effectively froze. We tried to dust them and bang the dust off and clean them, and there was just no way. The effect of dust on mirrors, cameras, and checklists is phenomenal. You have to live with it but you’re continually fighting the dust problem both outside and inside the spacecraft. Once you get inside the spacecraft, as much as you dust yourself, you start taking off the suits and you have dust on your hands and your face and you’re walking in it. You can be as careful in cleaning up as you want to, but it just sort of inhabits every nook and cranny in the spacecraft and every pore in your skin.”*

The surface lunar dust was lifted into the very thin lunar atmosphere as the astronauts walked over the lunar surface or drove their Lunar Roving Vehicle to explore the Moon’s surface. The surface lunar dust released into the lunar atmosphere by the astronauts resulted in dust-covered equipment, including space suits, helmets, scientific instrumentation, etc. (3).

An important and comprehensive report was compiled by NASA engineer James R. Gaier (4) on the impact of lunar dust on the Apollo astronauts, their health, and their equipment based on the Apollo mission reports, Apollo technical debriefings, and the transcripts of the voice traffic between the astronauts on the lunar surface and Mission Control. These documents, compiled as the

*Apollo Lunar Surface Journal,* are available online at http://www.hq.nasa.gov/history/alsj/. The Gaier report (4) should be required reading for the Artemis astronauts, mission planners, and engineers since, 50 y after the Apollo missions, there is a new generation of individuals in these professions, with little or no corporate memory of the devastating Apollo experience with lunar dust. Gaier divided the observed effects of lunar dust as described in the astronauts’ extensive postmission NASA debriefings into nine categories, which are reproduced here as direct quotes from the sources referenced (4, 5):

(1) Vision Obscuration: “The first dust-related problem experienced by the Apollo astronauts occurred when they landed the LM. The Apollo 11 crew reported that ‘Surface obscuration caused by blowing dust was apparent at 100 feet and became increasingly severe as the altitude decreased.’ This was even more of a problem for Apollo 12 where there was total obscuration in the last seconds before touchdown to the extent that there was concern that one of the landing feet could have landed on a boulder or in a small crater.”
(2) False Instrument Readings: “In Apollo 12 the landing velocity trackers gave false readings when they locked onto moving dust and debris during descent. The Apollo 15 crew also noted that landing radar outputs were affected at an altitude of about 30 feet by moving dust and debris.”
(3) Dust Coating and Contamination: “Dust was found to quickly and effectively coat all surfaces it came into contact with, including boots, gloves, suit legs, and hand tools.
(4) Loss of Traction: Neil Armstrong reported material adhering to his boot soles caused some tendency to slip on the ladder during ingress back to the LM.”
(5) Clogging of Mechanisms: “There were reports of equipment being clogged and mechanisms jammed on every Apollo mission. These included the equipment conveyor, lock buttons, camera equipment, and even the vacuum cleaner designed to clean off the dust. Dust made Velcro® fasteners inoperable and was a particular problem with some LRV indicator mechanisms.”
(6) Abrasion: “Lunar dust also proved to be particularly abrasive. Pete Conrad noted that the Apollo 12 suits were more worn after 8 h of surface activity than their training suits were after 100 h, and further reported that their EMUs [Extravehicular Mobility Units] had worn through the outer layer and into the Mylar multilayer insulation above the boot.”
(7) Thermal Control Problems: “An insulating layer of dust on radiator surfaces could not be removed and caused serious thermal control problems. On Apollo 12, temperatures measured at five different locations in the magnetometer were approximately 68 °F higher than expected because of lunar dust on the thermal control surfaces.”
(8) Seal Failures: “The ability of the pressure garment of the EMU to be resealed after Extravehicular Activitys (EVAs) was also compromised by dust on the suit seals. The Apollo 12 astronauts experienced higher than normal suit pressure decay due to dust in fittings.”
(9) Inhalation and Irritation: “Perhaps the most alarming possibility is the compromising of astronaut health from the irritation and inhalation of lunar dust. The Apollo crews reported that the dust gave off a distinctive, pungent odor (David Scott suggested it smelled a bit like gun powder), suggesting that there are reactive volatiles on the surface of the dust particles. Dust found its way into even the smallest openings, and when the Apollo 12 crew stripped off their clothes on the way back to Earth, they found that they were covered with it. Dust was also transferred to the Command Module during Apollo 12 and was an eye and lung irritant during the entire trip back. Given the potential toxicity of particle sizes less than about 10 μm, this points out the need to monitor the concentrations of dust particles within the EMU, the airlock, the habitat, and the spacecraft.”

Gaier (4) summarizes these findings with the following assessment: “Simple dust mitigation measures were sufficient to mitigate some problems like loss of traction, but for many such as thermal control problems, adhesion, and abrasion, it is clear that new capabilities must be developed for sustained lunar exploration. Some of these are also likely applicable for Mars. Some mitigation strategies, such as vibration, have been tried and found lacking. Others, such as brushing, appeared to work much better in ground tests than they did in the lunar environment. Clearly, an important area is the development of better simulation environments than were used in the Apollo era. This may include the use of better simulants, higher vacuum, more realistic thermal and illumination environments.”

## The Next Steps: Mars Dust, MSR, and Human Missions to Mars

As is the case for Earth’s Moon, Mars is a very dusty body, with atmospheric dust a regular feature of its atmosphere and responsible for its reddish/orange color (6). Estimates of the lifting of Mars surface dust into the atmosphere have been made for both a localized dust storm and a regional dust storm by the *Viking* Orbiters in 1977 (7). The mass of Mars dust lifted into the atmosphere associated with a localized dust storm near Solis Planum was 13 million metric tons (13 Tg) and the mass of atmospheric dust lifted into the atmosphere associated with a regional dust storm was 430 million metric tons (430 Tg). A recent estimate is that about 400 million metric tons (400 Tg) of dust is globally transported into the atmosphere of Mars every year and that the atmospheric dust settling rate produces a dust layer of about 50 to 100 μm on the surface of Mars (8).

As with prior robotic missions, future crewed missions to Mars must be designed taking into account what has already been learned about Martian dust and incorporating margins to account for what is not known about it. There are important questions remaining about this dust that, if answered, will result in fewer mission resources being applied against these uncertainties (6). These questions, difficult to answer with in situ instrumentation, may begin to be addressed by the MSR campaign and its returned samples and include:

1. What is the chemical composition and toxicity of dust transported globally by the atmosphere of Mars?
2. What is the particle size and shape distribution of dust in the atmosphere of Mars?
3. What are the electrical and magnetic properties of Mars atmospheric dust?

Another very important concern about Mars dust is its impact on PP compliance as outlined in next sections of this report.

## Human, Health, Safety, and Performance Considerations

On Earth, exposures to various types of dust or particulate matter (PM) can cause a range of health problems. International conferences on particle toxicities for humans started occurring in 1979, 7 y after the last Apollo Moon landing (9). Our understanding of the effects of PM on human health is still a developing field but a mechanistic understanding of pathologic processes associated with PM has advanced rapidly since the 1980s (9). Terrestrial industrial and occupational exposures to coal dust, asbestos fibers, and quartz/silica particles were the initial models of study due to an early association with both acute and long-term pulmonary health conditions (10). These exposures are known to cause pulmonary fibrosis and interstitial lung disease, mesothelioma, and some cancers. Outside of industrial exposures, particulates in cigarette smoke, urban air pollution, volcanic ash, and desert dust have all been linked to oxidative stress and inflammatory pathways within the body. These can include both acute respiratory issues as well as long-term health issues like pneumoconiosis, chronic inflammatory disease, silicosis, and interstitial disease. PM appears to cause not just pulmonary health issues, but also cardiovascular disease burden, skin irritation, and ocular irritation and allergic reactions as well.

Because of these historical health risks and a planned return to the Moon, NASA’s Office of the Chief Health and Medical Officer formally requested “…recommendations for defining risk criteria for human lunar dust exposure and a plan for the subsequent development of a lunar dust permissible exposure limit (PEL)” in the early 2000s (11). In 2005, the multicenter Lunar Airborne Dust Toxicology Assessment Group (LADTAG) held their first meeting to respond to this need and recommended research that would help experimentally elucidate the health risks astronauts could face from lunar dust exposure and recommend a PEL. Although expert consensus was not possible in 2005 because of significant unknowns about lunar dust at the time, the resulting research that was recommended and carried out eventually resulted in evidence-based Safe Exposure Estimate recommendations that were published in the 2014 LADTAG report (12). These included evaluations of ocular toxicity, allergic and immunologic response levels, and respiratory issues. Techniques for assessing toxicology of PM continue to advance, and some questions still remain regarding cardiovascular toxicity, surface reactivity, and variability of dust across the lunar surface. Dust from Apollo 14 was used by the LADTAG group because it provided intermediate physical and chemical characteristics between mare and highland dust but may not be representative of dust at the lunar south pole (12, 13). The research performed using samples enabled NASA to set an evidence-based atmospheric PEL for lunar dust particles of less than 10 µm in size of 0.3 mg/m^3^ over a 180-d period for habitats, spacecraft, and space suits, which is of significant relevance today as NASA embarks on the Artemis missions back to the Moon (13).

NASA’s Health and Medical Technical authority formally tracks a risk titled *Risk of Adverse Health and Performance Effects of Celestial Dust Exposure* (14). This risk encompasses health and performance issues that can occur as the result of lunar or Martian dust exposures and is still considered a “Red” risk (high concern) for a Mars mission (14). Information from the *Spirit* and *Curiosity* Rovers provides insight into particle sizes and chemical content and suggests that wind-blown dust across the Mars surface is likely homogenous, unlike subsurface particles (15–17). The *Spirit* Rover Microscopic Imager was limited to particle analysis down to 100 µm size (11). The dust has high salt levels, high concentrations of heavy metals and perchlorate, and high acidity (16). The perchlorate concentrations are concerning for toxicity with levels that are 3 to 4× more concentrated than typical soils on Earth (18). Perchlorate ions (-Cl0_4_) competitively inhibit the uptake of iodine in the thyroid gland and can affect hormonal function in humans. To date, ClO_4_- is the only Cl-oxyanion that has been found on Mars, but on Earth, it co-occurs with chlorate (ClO_3_-) in all environments and the health effects on humans are not well known. Ionizing radiation can also cause decomposition into other Cl-oxyanions ClO_2_- and ClO- which can cause skin burns, headaches, loss of consciousness, and vomiting for humans. The potentially corrosive effects of these chemicals on suit materials and instruments must be considered when informing repair and maintenance needs such as volume and mass needed for spare parts and tools. Davila et al. have recommended that research be performed with Martian samples to characterize the relative abundances of these Cl-oxyanions and better understand the potential impact on both human health and hardware (18). Electrostatic fields created in dust storms are likely to produce hydrogen peroxide which can condense, precipitate, and adsorb into the soil (19). For all these reasons and more, the NASA Evidence Report from 2014 concludes that Mars dust is likely toxic to humans (11). While this does not preclude human Mars missions, mitigations are likely to require special operational procedures or design features for safe exploration.

Mars samples could play a direct role in confirming and further characterizing the levels of toxicity that can be expected during the first human mission to Mars, and these data could then reduce design margin uncertainty or allow streamlining of operational workarounds, increasing the availability of one of the most valuable commodities in a human Mars mission: crew time. This research is helpful to inform an evidence-based PEL for the first Martian astronauts. However, it is not only a question of understanding the toxicities but also understanding how the seals, gaskets, instrumentation, and the Environmental Control and Life Support Systems (ECLSS) must be designed to ensure both a safe mission for humans from a toxicology standpoint and a safe mission from a reliability standpoint. Additionally, research on samples will provide guidance on medical countermeasures needed to deal with dust exposures and toxicity. This is important because as the next section notes, it is unlikely that we will be able to eradicate exposures completely. We are better prepared to return to the Moon for Artemis because of 9 y of research performed with lunar dust samples by the LADTAG program. A mission in the coming decade would provide the opportunity to ensure we have Martian samples that can be used to assess the effects of dust on toxicity and equipment wear so that we have strong, evidence-based information to provide to mission designers and engineers about the operational limits needed to protect future astronauts.

## Future Mission Design, Operations, and Resource Considerations

NASA has recently implemented a methodical process to coordinate and make meaningful progress toward planning for future human missions to Mars across the Agency. A description of this planning can be found in NASA’s Moon to Mars Architecture Definition Document (20). This process has not yet yielded a preferred architecture for these human missions nor has any decision been reached regarding where on the surface these missions would be conducted. However, there are known challenges related to the Mars environment—independent of how and where human missions are carried out—that must be addressed. This section discusses some of these issues and how MSR samples could help address them.

MSR samples would include small, lightweight “air fall” dust particles, and heavier local regolith particles. “Air fall” dust is important because it consists of particulates small enough to be suspended in the atmosphere and distributed planet-wide by mechanisms such as global dust storms. Consequently, this pervasive dust is thought to be compositionally similar across the planet (21) and can provide a “global” sample affording insight into the chemical, mineralogical, and physical characteristics of dust that would be found at any landing site on Mars. Returned regolith samples could also contain sand- and pebble-sized particles eroded from surrounding rock surfaces that are too heavy to be lofted into the atmosphere (particles that can be moved by saltation along the surface). Analysis of these particles might be used to gain a generalized (i.e., for other sites in addition to Jezero Crater) understanding of the local physical characteristics of these materials as they apply to the design and operations of surface elements, as well as In Situ Resource Utilization (ISRU) uses such as berm building or impacts on processing machinery creating commodities such as propellants (further discussion below). These analyses might also help to better calibrate orbital remote sensing instruments for more accurate identification of surface materials, including those considered useful resources for future human missions. Better data calibration could improve confidence in decisions based on these data—e.g., exploration site selection, when materials with certain chemical or mineralogical properties are important.

### Surface Design and Operations.

Much has already been learned about the engineering impacts of Mars dust and regolith from prior robotic landers and rovers, including quantitative results for the implications on solar power generation, and qualitative results related to the performance and reliability of mechanisms, e.g., wear rates and jamming frequencies. From a surface operations perspective, returned Martian samples would inform system design decisions for everything from exploration equipment to habitation design. For example, accumulation of dust poses a significant risk to power generation equipment, primarily solar panels, as was evidenced by NASA’s *InSight* Lander (22) and the *Spirit* and *Opportunity* Rovers (23). Because of its composition and fine particles, Martian dust can also impact seal designs on EVA space suits and tools, filters used to protect rotating machinery such as ISRU equipment of which the Mars Oxygen In-Situ Resource Utilization Experiment payload (24) on the *Perseverance* Rover mission is an example, and the ingress/egress methods of habitats and rovers, as noted from the Apollo experience. Dust samples would allow the designers of these systems to better understand and account for the dust’s impact on sealing and locking mechanisms. Returned samples would also enable a better understanding of the potential erosion of metal components that may come in regular contact with dust. For example, fasteners and bearings that are regularly exposed to dust may require more frequent maintenance due to erosion and degradation.

Despite best efforts to nominally control dust, contingency situations may result in dust entering a habitable volume, with implications to habitation systems including equipment seals, CO_2_ scrubbers, air filtration, and other components of the yet-to-be-designed ECLSS, as well as soft goods throughout the pressurized cabin. This may necessitate the inclusion of particulate air filtration systems sufficient to remove airborne dust, water filtration systems capable of removing water-soluble dust elements, and other internal system hardening to reduce the number of entry points for dust particles. The design of these filtration systems will need to be based upon either knowledge or assumptions about particle chemical/mineralogical composition and size distribution, with the former being much preferred.

Beyond the surface habitation elements, atmospheric dust is expected to affect crew landing and ascent, and surface mobility operations. Dust could obscure surface features—like craters, rocks, and abrupt changes in slope—and the easily movable dust can create uneven contact surfaces for vehicles of all types, any of which could pose hazards to mission equipment and operations. In order to avoid these possible hazards, landing sites will likely be examined prior to landing in a way that can account for dust accumulation. Analysis of returned dust samples might help to better characterize dust’s signature and differentiate it from other local surface material. Additionally, airborne dust, especially during dust storms, can reduce both the crew’s visibility during ascent and descent, and any line-of-sight navigation efforts that may be done from a surface rover or for telerobotic operations. Being able to design navigation components that can sufficiently penetrate airborne dust will be of value.

In all these examples—and similar cases for other surface equipment not mentioned—preparation of accurate simulants based on these returned samples would allow ground testing of candidate materials and potential design solutions to reduce design margin uncertainties.

### Resource Considerations.

Use of Martian resources has long been a recurring aspiration in plans for human Mars missions. It is still a factor in NASA planning, with the current “Moon to Mars Objectives” document (25) calling for characterization of Martian resources to enable eventual ISRU. Returned samples from Jezero Crater could provide valuable insights into methods for utilizing Martian regolith for propellant production, infrastructure improvements (e.g., berms, roads, etc.), and other uses.

Mars soil simulants are regolith analogs assembled using terrestrial constituents, based on data collected by orbiters and Mars surface rovers, such as those used in the development of Mars *Phoenix* and Mars Science Laboratory/*Curiosity* Rover missions (26). Simulants are widely used for research in a variety of Mars exploration disciplines. Examples include the following:

- Soil mechanical properties affecting rover wheel design or penetration methods for soil probes and drills
- Suitability of Mars soil for growing terrestrial crops, including biotoxicity and chemical mineral abundance
- Manufacturing and processing methods such as geopolymerization, sintering and resource extraction
- Electrostatic properties that affect how dust adheres to equipment, and processes for removal and cleaning
- Chemical reactivity testing for materials selection or ECLSS functionality
- Abrasive characteristics and the potential for wear on equipment, display screens and bearings, or degradation of seals and mating surfaces
- Response of terrestrial microorganisms to Mars soil as a PP consideration

In support of this research, simulant producers seek to replicate the chemical composition, mineralogy, and physical properties of Mars soil as accurately as possible. Returned regolith samples will better inform efforts to create higher fidelity simulants, giving higher confidence in analog research conclusions.

In addition to the analysis of the returned samples, lessons learned from the containment hardware and handling procedures for MSR samples can inform the development of the procedures and equipment used by human crews on Mars to protect both the scientific integrity of the samples and the safety of the crew. The multilayered containment system for MSR would “provide higher levels of isolation than anything achieved previously in space fight, including for the samples of lunar rocks, comet dust, asteroids, and the solar wind captured and returned to Earth by earlier NASA and international missions” (27). The results achieved by the containment system would provide useful input into the design of crewed Mars sample collection, containment, and transport.

## Planetary Protection Considerations

The United Nations Outer Space Treaty (28), to which the United States and all major spacefaring nations are signatories, lays out a consensus goal for the practice of PP. The NASA definition informing the U.S. implementation of these goals is stated as follows (29):

Planetary protection refers to the policy and practice of protecting current and future scientific investigations by limiting biological and relevant molecular contamination of other solar system bodies through exploration activities and protecting the Earth’s biosphere by avoiding harmful biological contamination carried on returning spacecraft, as described in the Outer Space Treaty. The main strategies are to:a. Understand and control harmful contamination of other worlds by terrestrial organisms, organic materials, and volatiles carried or released by spacecraft (referred to as forward contamination) in order to assure integrity in the search for evidence of extraterrestrial life and the study of prebiotic chemistry in the solar system for the appropriate period of biological exploration.b. Rigorously prevent harmful biological contamination of the Earth–Moon system by potential extraterrestrial life and bioactive molecules in returned samples from habitable worlds (referred to as backward contamination).

Within the practice of PP, several terms are commonly used to refer to the key concerns of the discipline:

Forward Contamination: The inadvertent transfer of Earth microorganisms to Mars that could potentially propagate and perturb potential indigenous life on Mars or confound future scientific measurements of Mars, in situ or via returned samples.

Backward Contamination: The inadvertent transfer of potential Mars microorganisms back to the Earth by the return spacecraft, equipment, instrumentation, or the astronauts themselves.

Round-trip Contamination: The case of forward contamination of Earth microorganisms into material being intentionally or unintentionally returned from Mars, such that it becomes confounding or ambiguous as to whether the discovered microorganisms ultimately originated from Earth or from Mars.

The challenges to future human missions to Mars posed by the principals of PP laid out in the UN Outer Space Treaty have long been recognized and been examined in numerous reports and workshops, e.g., refs. 30–32 and associated citations. Returned Martian samples could prove valuable in informing PP strategies, technologies, and protocols based on what is discovered from the initial samples returned. Whether these samples contain signs of present or ancient life, or materials of biological origin, or even organic materials, regardless of whether they can be shown to be of biological origin, would all inform and factor into approaches to PP for future missions, especially future crewed missions.

The site of the first MSR sample collection, Jezero Crater, was selected based on its interpretation as a paleolake with an intact and accessible deltaic fan, maximizing the ability of the *Perseverance* Rover to access exposed materials representative of a period in Martian geological history when conditions for life as we understand it were most favorable and where the conditions conducive to preservation of evidence of this life were greatest (33). Signs of ancient life in these samples would be a major scientific discovery, and immediately raise the question of the fate of these ancient life forms—do their descendants persist on the surface or subsurface of Mars today or if not, when and how did life on Mars cease to exist? Information from analysis of these samples would strongly inform future strategies for the exploration of Mars. Other sites and strategies have been identified that would have greater likelihood for the detection of extant life, but after much debate, the science community has reached consensus on the current overarching strategy of searching for signs of ancient life first, before investing further efforts in the search for extant life.

In the scenario of samples returned from the Mars 2020 landing site at Jezero crater on Mars, finding signs of extant life at a site selected with other priorities, while not expected, would inform the prospects of ubiquity of life at other sites across Mars. Even a negative or inconclusive result in the search for ancient life in the initial returned samples would be valuable in better informing the nature and composition of the regolith and other samples returned. Understanding the chemical and mineralogical composition of these and their potential to either nourish or repress the growth of life forms (of either Martian or terrestrial origins) is important to consider in the planning of future missions, especially those with crew. At a minimum, these missions will alter the sites that they explore thermally (by the mere presence of vehicles hosting crew habitats) but will also undoubtedly disturb the surface layers of regolith and expose underlying materials. Depending on how well spacesuits and other surface vehicles can be sealed, the possibility of release of gases, liquids, or even terrestrial biological materials/microbes may be possible or likely. Combined with new induced environments (increased temperatures, introduction of water/brines), the risks of forward propagation of Earth life to Mars (i.e., contamination) will be increased.

The risks of forward contamination of Mars may be either amplified or mitigated depending on the composition of the Martian regolith, which might be informed by the return of Martian samples and their analysis in terrestrial laboratories. While existing data strongly suggest that dust across the surface of Mars is currently well-mixed (21), owing primarily to aeolian transport, it is unknown the degree to which these same mechanisms would be effective in propagating forward contamination from future human missions to sites distant from the crew landing site but of astrobiological interest elsewhere, but this possibility has to be considered (34). On Earth, and potentially Mars, wind-blown atmospheric dust is a vehicle for the transport of microorganisms through the atmosphere. In addition, wind-blown atmospheric dust on Mars reduces the lethality experienced by potential Mars microorganisms from solar ultraviolet radiation. On Earth, microbial cells transported by atmospheric dust can travel thousands of miles and retain their viability (35). The typical airborne dust particle on Mars in “clear” skies (a typical dust optical depth or tau = 0.5) is in the size range of 1 to 2 µm. During a regional or global dust storm, the maximum particle size lofted can reach 8 µm. For comparison, on Earth, the typical size of a *Bacillus subtilis* spore is 1.07 µm × 0.48 µm, so in theory at least, clumps of spores could be dispersed. Further, during a typical regional or global dust storm, the UV flux can decrease by 95%, helping to protect the microbial cells and keeping them viable.

Size and composition distribution (e.g., either biocidal or protective factors), and covariances between these, coupled with atmospheric observations, would inform models of potential future microbial transport and likelihood that biological material carried by human missions would propagate or be carried and survive transport to other sites on Mars where they might subsequently become metabolically active and eventually able to replicate (36). Requirements on allowable forward contamination levels and the degree of rigor required on future crewed Mars missions would benefit from this information.

As mentioned previously, a special follow-on concern to the case of forward contamination is that of round-trip contamination, the “discovery” of living organisms within or on a vehicle returning from Mars. Again, the properties of Martian dust and regolith to exhibit either nutritive or biocidal characteristics is an important factor in informing the likelihood and potential mitigations to a round-trip concern, wherein organisms of terrestrial origin become mixed-in with Martian material being returned and create a confounding concern over whether these organisms are of terrestrial or Martian origin. Fortunately, recent work on the International Space Station demonstrating in-space microbial profiling utilizing nanopore sequencing techniques (32, 37) shows promise in providing a potentially powerful tool that could be used on future crewed missions to determine whether life forms detected in returned materials match DNA with inventories of terrestrial organisms known to be present on the outbound vehicle or if they represent novel organisms for which additional care must be exercised. Current nanopore sequencing technology requires hundreds of nanograms (ng) as input, obtained by collecting sufficient microbial biomass (e.g., from biologically rich material) or amplifying DNA with either nonspecific or targeted methods. Additionally, there has been recent work in demonstrating similar approaches in terrestrial labs based on low-biomass synthetic Mars analog soils (38). Detection of microorganisms sharing nucleic acid markers common with known terrestrial inventories would be in indication of concern for potential contamination but could also be interpreted as evidence of putative Martian life with some relation to life on Earth, the potential for which has been previously posited and considered (39–41).

## Conclusions

The understanding of the Martian environment that would be gained from sample return would be valuable in planning for future missions to Mars, especially those involving astronaut crew members. From the experiences of the Apollo Program, we now know what impacts extraterrestrial dust can have on the systems and crew we send to other bodies if we do not take appropriate precautions. By characterizing the elemental and mineralogical composition of Martian dust, as well as their particle size and shape distributions, and the covariance between these two characteristics, future missions can more appropriately plan their designs and operations based on this ground-truth. Absent the information that would be gained from actual samples, systems and operations would otherwise have to be designed with more bounding, conservative assumptions about Martian dust, which will consume precious resources and margins within what already promises to be the most ambitious spaceflight undertaking that is presently under consideration. The return of the first curated samples from Mars would not only provide an opportunity to better understand the history of Mars but could also reduce uncertainty in charting the future of humanity’s exploration of the planet.

## Data Availability

There are no data underlying this work.
